# Toys from childhood in immigration: placing memories into context

**DOI:** 10.3389/fpsyg.2025.1549330

**Published:** 2025-03-21

**Authors:** Ekaterina Protassova, Maria Yelenevskaya

**Affiliations:** ^1^Department of Languages, Faculty of Arts, University of Helsinki, Helsinki, Finland; ^2^Department of Humanities and Arts, Technion - Israel Institute of Technology, Haifa, Israel

**Keywords:** child development, creativity and imagination, family traditions, cultural identity, environmental concerns, intergenerational bonding, handmade vs. mass-produced toys, emotional well-being

## Abstract

**Introduction:**

Among the first objects which babies view and watch are various toys. The play has an enduring impact for the personal growth. The goal of our project was to explore why toys and playthings brought from the country of origin are an important component of the material and symbolic heritage in diasporic Russophone families, how their meaning is negotiated, and values assigned.

**Materials and methods:**

The study uses thematic, discourse, and content analysis drawing on a survey and group interviews. The participants (53 + 25), aged 5–61 at the time of relocation, emigrated from Russia and other post-Soviet countries to various destinations for personal, family, and professional reasons.

**Results and discussion:**

The results of the survey we conducted emphasize the multifaceted role of toys in child development, family culture, and education, stressing the importance of play over the toys themselves. Respondents advocate a thoughtful curation of toys to foster creativity and emotional growth, noting that an excess of toys may hinder children’s imagination. Psychologically, toys provide comfort, joy, and a sense of continuity amidst transitions. Families value traditional or handmade toys for their cultural and historical significance, creativity enhancement, emotional connections, and intergenerational bonding in the language transmission. The independent play fosters processing of emotions and transitions from life in a familiar milieu to a new environment. Environmental concerns about plastic toys and a preference for sustainable materials are raised. Participants view toys as contributors to children’s integration into the host society. The detailed personal narratives of those who migrated at an early age focus on the toys that shaped their early years revealing a profound significance of playthings, reflecting on the creativity and resourcefulness in their play and witnessing diverse economic circumstances and cultural backgrounds.

**Conclusion:**

Participants’ memories and experiences with toys show how language serves as a crucial medium for expressing and preserving memories, while culture provides the context within which these toys are understood and valued. Social interactions and cultural norms impact the significance of toys in the lives of the Russophone immigrants.

## Introduction

The most important features of toys for the earliest age are bright colors, melodious or funny sounds, and a pleasant touch. Then gradually toys become more sophisticated, and imitate objects which children encounter in real life. They discover how these objects function and learn their names. This can serve as an illustration that language does not exist without materiality. It swings back and forth in the social spaces between humans. Language is always used in social contexts (Pennycook, 2024). When we put language and materiality together at the center of analysis, we discover that a linguistic approach to materiality sheds light on processes of meaning-making and value production on the one hand, and incorporating materiality into linguistic analysis can ground such processes within social, cultural, political, and economic structures of power on the other hand (Shankar and Cavanaugh, 2017). A child’s understanding of an object’s purpose (its materiality) progresses from its life-sustaining and playful functions to the discovery of its primary and secondary meanings. This understanding is conceptualized both within the traditional culture at a certain historical stage of its development and in relation to specific family practices (Gabrys-Barker and Vetter, 2024). Materiality and language are connected in such a way that an object needs to be given a name, it should be describable, comparable to other objects, and classifiable. Depending on the criteria of classification, significant properties of the object are highlighted; these can be incorporated into the name as generic or individual features (Aronin and Melo-Pfeifer, 2023). Naive nomenclatures of the material world differ from scientific nomenclatures. And it is in the world of playthings that this naivety comes up strong as it reflects reality, but also phantasies and the world of make-belief. Naive nomenclatures are also reflected in everyday language, while each field of knowledge describes an object in its own way. Cultures impart special symbolic meanings to the object, and these may differ from one culture to another (Aronin et al., 2018).

Through retrospective analysis, researchers can show that participants’ memories of play reveal diverse definitions and experiences, aligning with dimensions of play, such as development, activities, and social and physical interactions. The study by Sandberg and Vuorinen (2008) highlights the sociocultural perspective of play, emphasizing how cultural values, prior experiences, and environmental factors shape individuals’ understanding and expression of play. Their research urges us to reflect on the evolving nature of play and growing media influences. They also show how adults’ perceptions of their children’s play can promote or hamper the children’s opportunities and willingness to explore, create, and process their daily experiences through play.

The study by Burr et al. (2019) used a phenomenological approach and Erikson’s psychosocial theory, gerotranscendence, and play theories to explore older adults’ reflections on childhood play and its role across the lifespan. Key themes revealed vivid memories of unstructured, risky, and physical play, contrasting with modern children’s highly structured, technology-driven, and “safe” play. Following Brussoni et al. (2015), we view risky play as exciting play that can include the possibility of physical injury. Different types of risky play include play at height, speed, near dangerous elements (e.g., water, fire), with dangerous tools, rough and tumble play (e.g., play fighting), and where there is the potential for disappearing or getting lost.

In many countries there are child injury prevention programs seeking to limit risky play to avoid physical injury. Societal and parental attitudes have encouraged increasing supervision and children’s diminishing independence in play. Today, restrictions of children’s opportunities for unstructured risky play are often discussed in terms of a potential negative impact on physical activity behaviors, development of alertness, and development of social skills (Whitebread et al., 2012). Making playgrounds safe and putting efforts in organizing children’s free time had an unexpected effect: children are highly confident in familiar settings (as most playgrounds today have more or less the same structure), but finding themselves in an outdoor environment which is not built-up in the same style, they tend to become bored and lack the imagination to invent and initiate games or play without a clear purpose (Quah, 2017).

Changes in children’s leisure activities have several reasons and different outcomes. First, there is a growing demand on children’s time at school. Parents and teachers prefer children to be monitored when they are outdoors, to be sure that they are safe. Unless rationed by the parents, the use of mobile devices takes the bulk of children’s free time. Moreover, in the increasingly urbanized world children have little opportunity to spend time in the nature. The results are reduced mobility and less range for exploration, including reduction in walking to school or riding a bike to school or to places of recreation. There is a growing fear of strangers, traffic and nature itself. Less physical activity leads to a dramatic rise in obesity and severe overweight, as well as vitamin D deficiency and other health issues that may in part be related to low levels of outdoor activity and a sedentary lifestyle (Charles and Louv, 2009).

Farley et al. (2022) explore how nostalgia and childhood innocence are constructed through everyday objects and play, revealing tensions between idealized notions of childhood and lived experiences, while calling for participants to reflect on and address the unequal vulnerabilities faced by children and educators. Ugaste (2023) examines childhood play memories across three generations in Estonia, highlighting the importance of freedom, variety, and outdoor play, which were associated with joy and excitement. Her findings reveal differences between urban and rural play experiences and showcase the wide repertoire of play across generations, dependent on the context of educational and political changes in Estonian society. The research by Babich (2014) demonstrates the tension between social conformity in educational institutions and the transformative, personal nature of play, emphasizing the need to reconcile dominant pedagogical theories of child development with intuitive, personal understandings of play in various social and educational contexts. It also highpoints contrasting views of play, where children see it as voluntary, free, and entertaining, while adults regard it as an educational tool, reflecting the influence of social structures and the dominance of social conformity in educational institutions.

Fairy tales, artisanal toys, and ecological traditions involving children in taking care of the plants and animals can reconnect children with nature, fostering sustainable values and behaviors through transformative learning processes that promote environmental stewardship and support local communities (Nath, 2023). The cultural, educational, and emotional significance of toys addresses their evolving roles in children’s lives, media practices, and societal contexts, from traditional dolls to digital and Internet-connected playthings. Many studies show that toys facilitate learning, self-expression, and contribute to inclusivity and media literacy. At the same time, they reflect deep social concerns about commercialization of toys and games, ambivalent influence of technologies on children and the way they play, and consumption-driven changes in cultural values related to upbringing. Over the past three decades, childhood has been transformed by the media and marketing industries. Marketers spend billions targeting children. Educators in some countries are aware of the dangers, so advertising targeting children is prohibited or limited. Commercialized childhood is unhealthy and ecologically unsustainable. Kids spending several hours a day watching children’s programs including overt and covert advertising, do not develop creativity and are left unprepared for a future that will require new kinds of behaviors, skills, and values (Golin and Campbell, 2017; von Schor, 2014).

The cultural-historical activity theory framework views play as an activity shaped by cultural history, involving the participation of others and thereby justifying adult involvement in play (Bodrova, 2008; Bodrova and Leong, 2010; van Oers, 2014; Vygotsky, 1997). For example, Loizou and Loizou (2022) define and contextualize creative play within the arts, highlighting the adult’s role as a cultural mediator and providing empirical data that support the enhancement of children’s creativity through strategic play involvement. Smirnova et al. (2016) claim that playground objects should encourage interaction between visitors, their surroundings, and each other, fostering intergenerational engagement. Hakkarainen et al. (2013) draw on Vygotsky’s concept of play, showing that adults need to become authentic partners in children’s play, employing suitable narrative techniques to establish the zone of proximal development for children. Devi (2022) advocates for active adult participation as play partners rather than passive observers, arguing that such involvement can support children’s problem-solving skills and abstract concept learning without dominating the play, as demonstrated by the findings from four Australian-Indian families.

Through diverse perspectives ranging from their role in family dynamics, gendered and racial positioning of subjectivities to social expectation around reading, sharing and demonstration of relative wealth, toys are much more than objects created for children. They allow us to analyze how they shape and are shaped by cultural, economic, and technological developments (Leaver et al., 2023). Moreover, many adults, including famous writers, are nostalgic for their toys and try to recreate happy moments of their childhood playing with their children and grandchildren, and toys play an important role in popular culture.

Our research questions in this project were:

- What types of toys do Russian-speaking immigrants bring with them when relocating, and what memories and emotional narratives are associated with these objects?
- How do the roles of toys differ across various age groups, regions of origin, and immigration timelines within Russophone immigrant communities?
- How does the material culture of toys impact emotional well-being, multicultural competence, and the preservation of cultural identity across generations in immigrant families?

The study which we conducted is praxis-related, so in the literature review which we presented in this section and our own views on the subject being discussed we focus on the research that looked at the practical implications of the significance of play and changes that have occurred in the approach to children’s upbringing in the last three decades.

## Materials and methods

Our study of material culture items applies thematic, discourse, and content analyses and is based on an online survey and group interviews to explore the significance of toys in the lives of Russian-speaking immigrants. Participants in the project were found in a snowball fashion, and no organization was involved in recruiting them. The questions of the survey were partly the same as the questions to the interviewees and they were initially discussed with our colleagues: educators, linguo-anthropologists and teachers of Russian as a heritage language. However, during the interviews new themes emerged thanks to the participants’ engagement and willingness to share their memories and opinions. So, we can say that our study has a contemporary historical perspective (cf. Sandberg and Vuorinen, 2008). We were not able to include all the questions and responses into one paper and made a specific selection for this one.

Besides reflective answers to questions, the interviews and survey responses included autobiographic narratives. The storytelling ability enhances individuals’ sense of meaning in life and endorses high-level goals with consistent results across diverse measures, narrative contexts, cultures, and personality types, showing the strongest effects among introverts. Narration of life stories increases self-esteem, which is particularly important for migrants many of whom face downward socio-economic mobility, at least in the first stages of life away from home (Einam et al., 2024; Singer et al., 2013; Steiner et al., 2019).

The 53 respondents in our online survey and 25 participants in the group interviews had diverse life paths, emigration years (from the 1990s to 2022), and ages (5–61 years) at the time of relocation, with an average emigration age of 30.35 years. The emigrants came primarily from Russia, including cities and regions such as Altai Area, Eastern Siberia, Irkutsk, Krasnoyarsk, Moscow and its region, Petrozavodsk, Saint Petersburg and its region, Samara, and Voronezh region. Others originated from former Soviet states, including Armenia, Estonia, Lithuania, Moldova, and Ukraine. Destinations included a wide array of countries: Austria, Croatia, the Czech Republic (Prague), Finland (Helsinki, Jyväskylä, Kuopio, Turku), France (Montpellier, Paris), Germany, Greece (Athens), Israel (Haifa, Petah Tikva, Tel Aviv), Italy (Milan, Rome), Luxembourg, the Netherlands, New Zealand, Spain (Tenerife, Valencia), Turkey, the United Kingdom, and the United States (California, New York, etc.), with some experiencing multiple relocations before settling. Their reasons for emigration varied from personal, family, and professional motives to escape from war-torn areas. Some moved due to a spouse’s new job, and some were led by an adventurous spirit. The interviewees live now in Finland, Germany, and Sweden. The questions in the survey and in the group interviews were rather similar.

In this study, informed consent was obtained from all participants who took part in the survey and in the interviews. Each participant was fully briefed on the aims of the study, their right to withdraw, and how the data obtained would be used. Written or oral consent was documented to ensure voluntary participation. All data collected were anonymized to protect participants’ identities. Pseudonyms were assigned, and identifying details such as names, locations, and specific references were removed or generalized. The anonymized data was securely stored in a password-protected system, with access restricted to authorized members of the research team. These measures ensured that privacy and confidentiality were maintained throughout the study.

Conducting thematic analysis, the process for identifying, analyzing, and reporting patterns or themes within data is recommended for projects in psychology, anthropology and culture studies (Braun and Clarke, 2006; Guest et al., 2012). We have applied it in our previous work and found it effective for singling out dominant motifs and classifying elicited data. Discourse analysis allows researchers to combine theory with practical examples to analyze language use in social contexts (Gee, 2014; Van Dijk, 1993). Qualitative content analysis offers practical advice and guides analysts in their search for the essence of what was said or written, be it explicitly or implicitly expressed (Krippendorff, 2019; Schreier, 2012). The combination of these approaches (Patton, 2014; Silverman, 2015) allows researchers to objectivize results of the survey and of the interviews.

### Results from the survey

In our previous studies (Protassova and Yelenevskaya, 2024a,b; Yelenevskaya and Protassova, 2023a,b), we examined various aspects of the connection between the past and present among Russian-speaking immigrants in different countries around the world. We explored their attitudes toward work, family, homemaking, food, cultural traditions, clothing, and entertainment. We identified their everyday linguistic and cultural practices, their home-making in the host countries, the objects that hold special value for them, and the memories they cherish. However, we have not yet considered which toys from their childhood retain significance after emigration. This project is our contribution to studies upon material culture of multilingualism.

#### Do you retain any memories of the toys and games from your childhood?

The overwhelming majority of respondents stated they had retained fond memories of their childhood toys and games, with some even keeping specific toys or recreating their favorite experiences as adults. Whether through preserving physical items, fondly reminiscing about interactive play, or seeking out lost treasures, these memories reflect the emotional value of childhood experiences. One respondent shared a memory of their mother’s celluloid doll, *Marinka* from 1930s, and the joy of receiving a rubber baby doll for her sixth birthday. Another noted a Japanese doll, highlighting the sentimental value tied to such an object. Some referenced traditional toys like a “cotton Snowman,” and “plastic Ded Moroz [Santa Claus],” showing a connection to their cultural roots and holidays. One respondent said the family cat nearly scalped her favorite doll *Alisa*, but she would not allow her parents to throw the doll out and replace it with a new one, because “Alisa was her ‘daughter’ and a bed companion.” To help Alisa recover from her trauma, her owner sewed a pretty dress for her.

One of the older participants wrote that her and her peers’ favorite place for play were stacks of firewood logs in the courtyard of her house in Leningrad. Back then there was no central heating in the house, and the firewood was stored for the winter. The children played hide-and-seek there, jumped from one stack to another (which she now admits was quite dangerous), and made “secrets”—digging into the ground colored pieces of glass, candy wraps with pictures and other “treasures.” Only the owners knew where their “secrets” were hidden and would regularly check whether the treasures were still there, admired them and added new objects. These games emphasize social interaction and teamwork and reflect how play could help build friendships and memories.

Several respondents either still own their childhood toys or have sought them out as adults, showcasing the lasting emotional impact. For example, one person purchased her first doll on a resale platform to rekindle childhood memories. A few mentioned that toys were scarce during their childhood, which made them treasure the few they had. Others recalled entertaining themselves by playing creative games like “word games on paper” or “playing dollhouses in furniture drawers.”

One respondent recalled how she had played “theater” with her friend. They used two toy animals, a fox and a rooster. The two of them had a difficult relationship, both trying to outsmart the other. The dialog was invented on the spot and included themes from fairy tales and reflections from the girls’ everyday life.

#### Do you remember any board games from your childhood? Are they played today? What board games do you play now?

The responses reflect a broad spectrum of experiences with board games, ranging from nostalgic memories of traditional games to engagement with modern and strategic games. Many respondents fondly remember classic games such as lotto, dominoes, chess, and checkers. Popular among them are games like *Monopoly, Mafia*, memory-matching games (e.g., *Memory* or *Pexeso*), and simpler games involving dice and moving pieces, such as *Snakes and Ladders* and its variations. Some recall unique games from their childhood, like *Fox and Geese* or *Circus*. A significant number of respondents noted that their families play modern board games today. Families also play educational and logic games like *Ubongo, IQ Love*, and *Rush Hour*, emphasizing development of cognitive and strategic skills. Many play with children and grandchildren, often choosing games that are educational or promote memory and language skills, such as *Spot It, Dixit*, and *Lotto*. Respondents highlighted that board games foster family bonding and serve as a way to pass traditions down to younger generations. Some families have created their own board games or modified existing ones. For example, one respondent mentioned sewing felt-based board games for her children, while another noted that kids often invent their own rules for existing games, adding creativity to traditional play. Despite this, efforts to introduce older games to younger generations were evident, with some respondents bringing games from Russia or adapting them to suit bilingual households.

In sum, board games remain a beloved part of family and personal traditions, bridging the gap between generations. While classic games like lotto, dominoes, and *Monopoly* continue to be played, modern families also embrace newer games, which cater to diverse interests and skill levels. Board games not only evoke nostalgia but also serve as tools for cognitive and linguistic education, creativity, and bonding, evolving with time to remain relevant and cherished.

#### Do you play chess? Where is your chess set from?

Chess is a specific phenomenon, and bilingual families in our previous research often mentioned that they want their children to play it. The responses highlight diverse attitudes toward chess, reflecting its cultural significance, varying interest levels, and the sentimental value of chess sets. Some respondents actively play chess, either casually or with family members. Several participants noted that their children or spouses were serious players, with one family even having a member who trains with a chess coach. Respondents own souvenir sets from countries like Greece, Bulgaria, or Vietnam; handcrafted or designer sets, such as wooden sets from Germany or replicas of historical designs. Miniature or travel sets were highlighted as practical for enthusiasts who travel frequently. Several participants mentioned knowing the basics but not playing regularly. Reasons include limited time for leisure, a lack of interest in the game among other family members, and perception of chess as too challenging a game for relaxation during leisure hours. Some respondents noted regional variations in chess-like games, such as Italian checkers which differ in rules from Russian ones. All in all, chess remains a cherished activity for many, either as a competitive game or a nostalgic link to family traditions.

#### Do you play “*Erudit*” (*Scrabble*) or other word games? What language do you play in?

Responses reflect a wide range of word-game preferences and practices. Many respondents enjoy *Scrabble/Erudit* (alternative Russian name), with some playing on physical boards and others using apps. Other word games like *Goroda* (Cities), *Viselica* (Hangman), and apps like *Wordscapes* or *Filwordy* are popular. Participants frequently mentioned educational word games for young children, e.g., a series by “Banda Umnikov” (smart guys gang). These games aim to develop memory and attention, expand children’s vocabulary in different domains, e.g., animal world, human body, outer-space and others, and learning to read and count in a dynamic and entertaining way. Other specific games include *Alias, Bulls and Cows*, and *Crocodile* (a charades-style game).

Several noted challenges when playing in non-native languages, especially for children or learners. Language choice often depends on the players’ linguistic abilities. Families frequently play in Russian, especially when teaching children the language. Some mix languages depending on the group or game. Several respondents shared memories of playing *Scrabble* or similar games during their childhood, often associating them with learning or family time. Some have not played due to children’s age (e.g., “not yet reading”), lack of time, or difficulty acquiring a set in their current location.

#### How do the games of today’s children differ from the games of your childhood? Has the role of games changed today?

A prominent theme is the shift from outdoor and physical games to digital and screen-based games, with many parents and participants noting the dominance of gadgets such as tablets, smartphones, and video games. Traditional games required more creativity, as children often improvised with available materials (e.g., making doll furniture from boxes, using sticks as props, and sand, water, leaves and grass when playing a grocery store). A participant observed, “Now there is less room for imagination; everything comes with instructions.” Today children have access to a broader variety of toys and games, including advanced educational tools, Lego, and themed sets. However, some respondents remarked that toys today often feel “disposable” or lack emotional and creative value; moreover, they give fewer opportunities for group play: in the past, children played more communal games such as hide-and-seek or tag, often without adult supervision. Today, games have become more individualistic or require parental involvement. As one respondent stated, “Games today often involve a child and a toy or gadget, rather than children playing together.” A decline in neighborhood or “street” culture was also noted, with traditional group games like *Cossacks and Robbers*, *Shtandart*, or *Hopscotch* fading from popularity.

Our participants pointed to the educational and competitive focus of contemporary games which are often designed with clear developmental or competitive goals. For example, Lego sets teach spatial skills, and video games challenge reflexes and teach problem-solving skills. A respondent highlighted this shift: “Children are motivated by the recognition they get for excelling at games.” Some participants criticized digital games for limiting active engagement and imagination, reducing the child’s role to “pressing buttons.” Parents are now more involved in structuring and supervising children’s play. This contrasts with past generations, where children played independently and learned from peer interactions.

#### How do modern toys differ from toys of your childhood?

The responses to this question reveal several recurring themes, showcasing a broad spectrum of opinions about the evolution of toys. Many respondents highlighted the sheer abundance and variety of modern toys compared to their childhood. Phrases like “enormous assortment,” “more options for development,” and “infinite variety” were frequently used. This reflects how the commercialization of toys has grown, providing children and parents with an unprecedented range of choices. Several respondents mentioned that the ease of access to toys has led to their diminished value in the eyes of children. One participant noted, “Our toys were few and therefore cherished.” Modern toys are often equipped with advanced features such as lights, sounds, and even artificial intelligence. Respondents describe these as “smart,” “technological,” and “interactive.” This evolution has made toys more engaging but has also raised concerns about their impact on creativity. Comments like “total digitalization” and “abundance of electronic games” emphasize the influence of technology on toy design, steering away from the simplicity of older toys. Many noted that modern toys are more visually appealing with brighter colors and detailed designs, however, with “unrealistic proportions such as eyes that are too big” and “fluorescent, aggressive designs.” The prevalence of toys inspired by movies, cartoons, and pop culture was frequently mentioned, reflecting how media influences children’s preferences.

Respondents lamented that modern toys often come with specific instructions or predesigned uses, reducing the scope for creativity. Comments like “everything is already thought out for the child” and “no need to use imagination” illustrate this concern. Many responses praised the educational value of modern toys, describing them as “smart,” “developmental,” and “helping speech and logic.” The rise of STEM (science, technology, engineering, and math) and creative kits reflects this trend. The extensive use of plastic and its environmental implications were noted, with comments about “toys breaking easily” or being “designed for one-time use.” Respondents reminisced about the sturdiness and sentimental value of older toys, which were often handmade or passed down.

#### What’s important in children’s toys?

The responses to this topic reflect a deep consideration of the role of toys in child development, family culture, and education, as well as strategies for selecting toys based on a child’s individuality. Comments like “toys are not that important if the child plays” and “toys should support play rather than replace it” reflect a belief that creativity and imagination are central. Several responses noted that too many toys can stifle creativity. Phrases like “there should not be too many or too few toys” and “a clear hierarchy of favorites among toys is necessary” suggest the need for thoughtful curation.

Many families create toys together, which fosters creativity and emotional connection to the self-made objects. Examples included making toys from natural materials like cones and leaves or restoring old dolls. One respondent described how this process adds depth, as handmade toys are often seen as “alive” compared to store-bought ones. Some responses highlighted the significance of traditional or old-fashioned toys, noting that they carry stories and historical value, often passed through generations. Examples included repairing Soviet-era dolls and incorporating storytelling into play. Several participants mentioned the importance of toys that reflect cultural heritage, such as *matryoshkas* or other traditional Russian toys. While some families prioritize this, others instill cultural identity through literature and cinema instead. Responses stressed traditions like decorating homes with vintage or handmade ornaments, as well as celebrating holidays with games that unite generations (e.g., lotto during the New Year Eve).

Several respondents described using toys to process emotions, particularly after stressful events. One noted how “children use play to calm down after returning home.” Toys are seen as tools to enhance creativity, problem-solving, and emotional intelligence. Respondents described fostering role-play and scenarios with “human-like toys and animals” to encourage storytelling and emotional processing. Some noted the importance of games like *Zaitsev cubes* (an alphabetization set) and other educational tools that promote learning literacy or math through play. Comments like “plastic toys are too many and dangerous” highlight the need for more sustainable materials; several participants criticized the overuse of plastic, citing environmental concerns and safety risks. One respondent described creating meaningful connections by associating specific toys (e.g., rubber ducks) with family members.

Participants suggested a range of additional questions, such as: How do families control the number of toys in the home? Do parents encourage gender-neutral play or have specific “toy policies”? What role do traditional/national toys play in modern childhood? How do toys contribute to intergenerational bonding or the preservation of family traditions?

### Former immigrant children reflect on the toys that shaped their childhood

The discussion reveals that the value of toys extends beyond the physical objects, encompassing creativity, emotional connection, family involvement, cultural identity, and sustainability. Toys play a crucial role in child development, family culture, and education, fostering creativity, transgenerational communication, and emotional growth.

#### The value of toys in play and creativity

The consensus among respondents is that the true value of toys lies in their ability to inspire play, creativity, and invention. R1 and R3 recall the significance of their toys, such as R1’s puppet from “Spokoinoy nochi, malyshi [Good night, small ones]” and R3’s comfort in a pink plush cat. R1 shares a poignant memory of longing for a Baby Born doll, which she later fulfilled for her sister. R7 adapted a Transformer toy as Barbie’s partner, demonstrating creativity. R16 and R22 used everyday objects in imaginative play, like nail polish bottles and a musical toy.

#### Emotional and sentimental value

Toys often hold deep emotional significance. R2 cherishes a plush animal from her grandmother, while R3 finds comfort in a plush cat bought during a tough period. R15 received a soft mammoth from her best friend, and R19 fondly remembers a simple plastic doll named *Alyoshka*. R10’s favorite toy is a pink pig named *Pavel*, which she sleeps with every night. R12 and R13 have kept favorite childhood toys, like rabbits and a plush dog. Overall, plush animals are often the most cherished toys imported when moving from one country to another.

#### Family involvement and cultural identity

Family involvement in toy creation and selection is crucial. R2 and R25 highlight the emotional role of handmade toys, with R25 making dollhouse furniture with her great-grandmother. R1’s father created a customized board game, and R7’s family preserved toys for future generations. Traditional toys like matryoshkas and Soviet-era items carry cultural significance, as noted by R17 and R8. R24 and R20 recall toys that connected them to family members.

#### Non-interference in play

Respondents emphasize the importance of allowing children to direct their own play. R16 and R22 recall imaginative play without adult interference, using toys to process emotions and cope with border transitions. This autonomy fosters emotional and cognitive development.

#### Sustainability and environmental concerns

Many respondents criticize the overuse of plastic toys, advocating for more sustainable and safer materials. R9 and R4 appreciate the durability of older toys, while R8 mentions the environmental impact of modern, disposable designs. R13 and R6 appreciate toys with sentimental value over mass-produced items.

#### Favorite board and strategy games

Board and strategy games are cherished for fostering creativity and bonding. R1, R5, and R6 mention playing chess, checkers, and card games. R1 recalls a customized board game based on Russian folklore, while R7 and R4 highlight the cultural influences in their games, like “The African Star” (the most famous Finnish board game) and say, “Well, I bought these Soviet dominoes again.”

#### Toys as heirlooms and family bonds

Passing toys through generations preserves memories and family history. R7’s original Barbies are enjoyed by her niece, and R20’s rubber penguins remain in his mother’s storage room. R9 mentions a family heirloom, a large glass Christmas ornament. R22’s musical toy from the Tchaikovsky House Museum is cherished but fragile.

As we see from the results, there are significant differences between respondents who immigrated in the 1990–2000s and those who immigrated more recently. One might hypothesize that more recent Russian immigrants had play experiences that were more similar to their Western peers compared to those whose childhood years were closer to the Soviet era. This expectation is grounded in the historical and cultural shifts that have occurred in Russia and other post-Soviet countries over the past few decades. During the Soviet era, children’s play experiences were heavily influenced by limited consumer goods, and a distinct cultural environment (cf. Rumyantsev, 2025). Toys were often standardized and designed to promote collective values and ideological education. In contrast, the post-Soviet period saw greater exposure to global markets, increased availability of diverse toys, and a shift toward more individualistic and consumer-oriented play experiences. By comparing these two groups, we uncovered how the changing sociopolitical landscape and cultural integration influenced the nature of childhood play and the significance of toys. The evolving identity and cultural assimilation of Russian-speaking immigrants were prepared better than before; however, historical context, cultural background, and personal experiences might not be in favor of the integration.

## Discussion

The fondness for vintage toys, evoking childhood memories, is common across various ages and cultures (e.g., Baker, 2023; Kostiuhina, 2008; comments around the toy museums; internet forums and blogs dedicated to nostalgic dolls, cars, trains, games etc.). Comparing the memories of children who lived between two cultures with those who had a monolingual childhood, we notice that they associated different life zones (places) with specific toys and languages (where and what they played, to whom and what they gave, which opportunities were connected with which countries). As researchers of multilingualism and multiculturalism, it is important for us to note that the interaction of language with space is one of the markers of identification. Consequently, the materiality of multilingualism was already being formed in childhood.

The theme of toys and play interests researchers specializing in psychology, sociology, anthropology, pedagogy, educators and parents. One of the reasons is that professionals and lay people alike are concerned about the consequences of commercialization of childhood and excessive regulation of children’s time spending. Russophone living in Russia and diasporans are no exception. Thus, in the Russian segment of the internet we find numerous sites discussing games of the past and of today. We find sites which give detailed descriptions of court-yard games which were popular in the Soviet times but are unfamiliar to children of today many of whom spend most of the time with gadgets. The authors emphasize the freedom that children enjoyed in choosing how to play, and the impact these games had on developing physical strength and agility, as well as development of team spirit and creativity^1^^,^^2^. Émigrés, on the other hand, are mostly interested in discussing games for speech development of young bilinguals. Most of the contributors in the forums are Russian-language instructors specializing in teaching heritage speakers, but parents also participate proposing their own versions of games for vocabulary acquisition, pronunciation practice and grammar learning (see, e.g., Facebook group *We play with Vika. Early development. Bilingualism. Speech therapy*. The group was created by Vika Raskina and numbers 53.1 K members) and *Kaleidoscope school* for on- and offline classes, consultations for parents and testing bilinguals to determine their level of proficiency in Russian https://schoolkaleidoscope.com/o-shkole. On the website of the school users can find descriptions of games. Although they claim their target is bi- and multilingual children, they do not really show how different languages in the child’s repertoire are utilized. Neither do they mention the principle of building up parallel vocabularies as a means to achieve balanced bilingualism.

Toys can bring families together through shared play, whether through storytelling, team games, or holiday traditions. In some cases meaningful connections are created by associating specific toys with family members. Several respondents emphasized the sentimental value of passing toys through generations, preserving memories and family history. Cultural identity and heritage emerge as significant motifs, with respondents noting the value of traditional or handmade toys that carry historical significance. Toys like matryoshkas and other traditional Russian items are cherished for their cultural background, which becomes particularly important when people relocate. Stories about old toys are often passed down through generations. While some families prioritize these traditional toys, others instill cultural identity through literature and cinema, reflecting diverse approaches to cultural education.

The theme of non-interference in children’s play is also prevalent. Comments like “it’s important not to dictate what to play” highlight the belief that children should have the freedom to direct their own play, fostering independence and creativity. This autonomy is crucial for emotional and cognitive development, as children use play to sort out their experiences, process their emotions and navigate transitions, particularly after stressful events. Notably, forums for parents also warn against excessive control and establishment of strict rules but rather advising negotiation of the rules with the children^3^.

As Veraksa et al. (2020) formulate, childhood play is a complex evolving phenomenon, which takes various forms to support development and education, yet research in this field faces methodological challenges due to diverse theoretical perspectives and the lack of a unified classification. Many stressed that parents should not interfere with play unless invited, with comments like “it’s important not to dictate what to play and how.” Allowing children the freedom to direct their play fosters independence and creativity. Many respondents emphasized that the value lies in the act of playing, not the toys themselves. Making toys together unites the family and fosters communication of specific type. Respondents praised older, sturdier toys and expressed dissatisfaction with modern, disposable designs. Toys can bring families together through shared play, whether through storytelling, team games, or holiday traditions (cf. Bodrova et al., 2023).

Many participants in our project emphasized the value of playing over the type of toys available for games. Both the interviews and the survey reveal that while toys are important, their true value lies in their ability to support creativity, emotional growth, and family connections. Thoughtful selection, moderation, and an emphasis on play over possession are key strategies for choosing toys that align with a child’s individuality and developmental needs. The variety of toys mentioned or described by the respondents is immense, and there is consensus as to which ones were particularly important when families relocated (stuffed animals, favorite dolls, Christmas tree decorations and paraphernalia for role play).

A salient theme is comparison of mass-produced toys to those created by family members together, which fosters creativity and emotional connection to the self-made objects. Sustainability and environmental concerns are recurring themes, with many respondents criticizing the overuse of plastic. Statements like “plastic toys are too many and dangerous” reflect a collective desire for more sustainable and safer materials. Older, sturdier toys are praised for their durability, and there is a clear dissatisfaction with modern, disposable designs. This is a notable motif, because until recently, Russophones were rather indifferent to ecological issues (cf. Murphy, 2019).

Although there were few explicit statements concerning gender differences in play, it is clear that traditional gender roles are reproduced in games and toy selection. Girls like dolls, primarily those representing girls. They like to dress them and value clothes that are bought for their Barbies. They also love to play with miniature furniture, decorating their dolls’ “apartments.” They use water, grass and leaves to cook for their favorites. The boys are in love with cars, trucks and machinery, as well as toy weapons, which worries some of the parents.

The data we analyzed testifies that fascination by toys and play are not restricted to childhood and adolescence. Some adults are no less passionate than their offspring when they play together. Others value an opportunity to acquire toys they could not get in their childhood. And some young adults aware of their parents’ or siblings’ attraction to some toys that were lost, broken or left behind when the family migrated try to buy replacements for the cherished and nostalgic objects.

Adults reflect on the evolution of toys noting that modern toys are more diverse, technologically advanced, vibrant, and abundant (cf. Veresov and Veraksa, 2022). Many toys now have digital features, electronic effects, and are tied to media franchises (cartoons and movies). Some parents resent aggressive advertising of toys compelling their children to compete with their local peers from more affluent families. They observe that in their childhood toys were simpler, fewer in number, and more cherished. They often required imagination to play with, unlike modern toys that are often “ready-made” and prescriptive in use. A dominant motif in the elicited data is that the act of play is more important than the toys themselves. Creativity, imagination, and emotional expression are central to meaningful play. Too many toys can overwhelm and stifle creativity, while too few might limit opportunities for variations in play (cf. Henrich, 2014).

As already mentioned, integrating cultural traditions and considering sustainability are also crucial factors in toy selection. The collective insights underscore the multifaceted role of materiality in enriching childhood experiences and fostering lasting family bonds. As we see, the childhood memories of former immigrant children reveal the profound impact toys had on their lives, serving as reflections of their unique experiences (e.g., toys’ names) and cultural backgrounds. From cherished plush toys and handmade dolls to longing for unattainable toys like Baby Born dolls and Bratz dolls, these narratives emphasize the emotional significance and sentimental value attached to their playthings. The stories also underscore the creativity and resourcefulness in the children’s play, whether through custom-made board games, imaginative outdoor activities, or inventive use of everyday items (cf. Chang-Kredl et al., 2024). Despite varied economic circumstances and cultural influences, toys provided comfort, joy, and a sense of continuity, bridging their past and present. These recollections not only point out the universal nature of play but also demonstrate how toys can serve as enduring symbols of childhood, family bonds, and personal growth.

## Conclusion

This study examines the multifaceted role of toys in child development, family culture, and education, emphasizing that play itself is more important than toys whatever memories are connected with them. Respondents advocate the thoughtful curation of toys to foster creativity, emotional growth, and independence, while cautioning that an excess of toys may stifle imagination. Many families engage in creating handmade toys, enhancing emotional bonds and valuing their cultural and historical significance. Family members often create toys together, trying to enhance children’s creativity and intergenerational emotional connections. Handmade toys are often valued as more “alive” and meaningful compared to the mass-produced ones. Traditional or cultural toys, such as *matryoshkas, Baba Yaga* or characters from children’s books, such as *Vasilisa-the-Beautiful*, *Buratino, Cheburashka* and others, as well as renovated dolls, carry historical and familial significance. Moreover, older toys were often sturdier and were passed on from older family members to the next generations. Sometimes there were stories connected to them, and so they have sentimental value that has grown more powerful due to the traumas of relocation. Children learn to be careful when using them.

Some of the narratives reveal that adults may still want to play with toys. In some cases, this is a compensatory mechanism when they can afford and acquire the toys of their childhood dreams which were unavailable for them. Some others try to overcome nostalgia for happy childhood when they did not have responsibilities and were not confronted with moral, social and economic problems. Many adults love puzzles and find it a relaxing but also a challenging activity. Some parents are equally fascinated by the newest toys incorporating digital technologies and sometimes they compete with their children playing with electronic toys.

Additionally, the second part of the research explores the childhood memories of immigrants who relocated as children, revealing the emotional and cultural importance of toys in providing comfort, joy, and a sense of continuity amidst economic and social transitions, when immigrant children intuitively feel the stress of the parents. The findings underscore the universal nature of play and the enduring impact of toys as symbols of creativity, family connections, and personal growth across generations and cultural contexts.

Contemporary toys are sometimes seen as disposable or overly commercialized. The study also raises environmental concerns about plastic toys, expressing a preference for sustainable materials, and mentions the role of toys and games in intergenerational bonding and preserving family traditions. Toys can reflect ethnic and cultural identity, but some families prioritize cultural transmission through books, films, or storytelling instead of toys.

Holiday traditions involving toys (e.g., decorating fir-trees with vintage ornaments, family board games like lotto) were highlighted as bonding experiences. Participants in the project emphasized that adults should support children’s play without controlling it, allowing children to explore independently. Toys can help children process emotions and turbulences, with parents acting as facilitators rather than supervisors. Toys can support learning and skill-building, from literacy games to role-play scenarios that develop emotional intelligence and storytelling skills. Some participants complain that there are toys representing animals or characters from movies and cartoons which look aggressive or scary and can inhibit children, so they try to avoid buying them. Intergenerational connections were highlighted, such as restoring old toys or continuing family traditions around play.

Russian-speaking immigrants often bring toys that hold deep emotional significance, such as handmade items, playthings cherished by parents or their children. Gifts from loved ones, evoking memories of home, comfort, and continuity amidst relocation acquire special value away from the former country of dwelling. The roles of these toys vary across age groups and timelines: for children, they provide familiarity and stability; for adults, they symbolize nostalgia and cultural heritage. Regional origins influence the types of toys valued, reflecting local traditions and resources. Overall, the material culture of toys supports emotional well-being, fosters multicultural competence by blending cultural influences, and aids in preserving cultural identity across generations within immigrant families.

## Data Availability

The anonymized data supporting the conclusions of this article will be made available by the authors upon request.

## Footnotes

^1^ https://vm.ru/society/320498-25-dvorovyh-igr-nashego-detstva-kotorye-sejchas-malo-kto-pomnit

^2^ https://arzamas.academy/materials/1341

^3^ https://n-e-n.ru/detskie-igri-v-mirnoe-ruslo
